# Beyond Missing Heritability: Prediction of Complex Traits

**DOI:** 10.1371/journal.pgen.1002051

**Published:** 2011-04-28

**Authors:** Robert Makowsky, Nicholas M. Pajewski, Yann C. Klimentidis, Ana I. Vazquez, Christine W. Duarte, David B. Allison, Gustavo de los Campos

**Affiliations:** Department of Biostatistics, University of Alabama at Birmingham, Birmingham, Alabama, United States of America; Georgia Institute of Technology, United States of America

## Abstract

Despite rapid advances in genomic technology, our ability to account for phenotypic variation using genetic information remains limited for many traits. This has unfortunately resulted in limited application of genetic data towards preventive and personalized medicine, one of the primary impetuses of genome-wide association studies. Recently, a large proportion of the “missing heritability” for human height was statistically explained by modeling thousands of single nucleotide polymorphisms concurrently. However, it is currently unclear how gains in explained genetic variance will translate to the prediction of yet-to-be observed phenotypes. Using data from the Framingham Heart Study, we explore the genomic prediction of human height in training and validation samples while varying the statistical approach used, the number of SNPs included in the model, the validation scheme, and the number of subjects used to train the model. In our training datasets, we are able to explain a large proportion of the variation in height (*h^2^* up to 0.83, *R^2^* up to 0.96). However, the proportion of variance accounted for in validation samples is much smaller (ranging from 0.15 to 0.36 depending on the degree of familial information used in the training dataset). While such *R^2^* values vastly exceed what has been previously reported using a reduced number of pre-selected markers (<0.10), given the heritability of the trait (∼0.80), substantial room for improvement remains.

## Author Summary

While previous genome-wide association studies have implicated numerous loci associated with complex traits, such loci typically account for a very small proportion of phenotypic variation. However, a recent study using height as a model trait has illustrated that common single nucleotide polymorphisms can explain a large amount of genetic variance when evaluated through whole-genome statistical models. However, it is unclear to what extent higher proportions of explained variance will translate into improved predictive accuracy in future populations. Here we evaluate the predictive ability of whole-genome models for human height while varying the modeling approach, the size of the training population, the validation design, and the number of SNPs. Our results suggest that whole-genome prediction models can yield higher accuracy than what is commonly attained by models based on a few selected SNPs; yet, given the heritability of the trait in question, there exists room for improving prediction accuracy. While gains in predictive accuracy are likely to be small based on more expansive genotyping, our results indicate that more substantial benefits are likely to be gained through larger training populations, as well through the inclusion of related individuals.

## Introduction

Few examples exist of findings from Genome Wide Association Studies (GWAS) being applied to preventive and personalized medicine. Despite the success of GWAS in the discovery of many novel disease variants, the variants identified as being statistically significant typically account for minimal fractions of the genetic variance, even for highly heritable traits [1]. This so-called “missing heritability” has prompted a wide array of explanations, ranging from poor modeling (e.g., unaccounted epistatic effects) [2], [3], insufficient sample sizes [4], sparse genetic coverage [5], rare variants [6], undetected CNV effects [7], and over-estimated heritability [1], [8], [9]. While all of these problems (and possibly others [10]) likely contribute to some extent [11], recent articles by Yang *et al.* [12] (hereafter, the Yang Study), and others [13], [14] suggest that the primary culprit may be a mismatch between the actual genetic architecture and the statistical techniques applied.

Typically, predictive models from GWAS are constructed using a small number of Single Nucleotide Polymorphisms (SNPs) that have been pre-selected using extremely low p-values derived from single-marker regressions. This approach is most sensible under the assumption that only a few loci affect the trait of interest; however, it performs poorly for complex traits [14], [15], which could be subtly affected by many loci [16]. Drawing on methods commonly used in animal breeding [17], the Yang Study built a model for human height (a model trait that has recently received much attention because of its high heritability and relatively reliable phenotyping) with hundreds of thousands of SNPs jointly considered (see Visscher et al. [18] for an expanded commentary on the methodology employed).

Using a Whole Genome Prediction (WGP) method, the authors from the Yang Study estimated that common SNP variation (through Linkage Disequilibrium (LD) with causal polymorphisms) explained 45% of the phenotypic variance, thus accounting for more than 50% of the expected heritability of height, which is reported to be approximately 80% [19], [20]. These results suggest that the underlying genetic architecture of human height likely consists of numerous polymorphisms of small effect, resembling the infinitesimal model of quantitative genetics [21], [22]. Recent studies suggest similar conclusions for other complex traits, including schizophrenia and bipolar disorder [23], blood lipid levels [24], and body mass index [25], suggesting a broader utility for the approach of WGP methods to account for genetic variance of important complex human traits.

**Figure 1:**
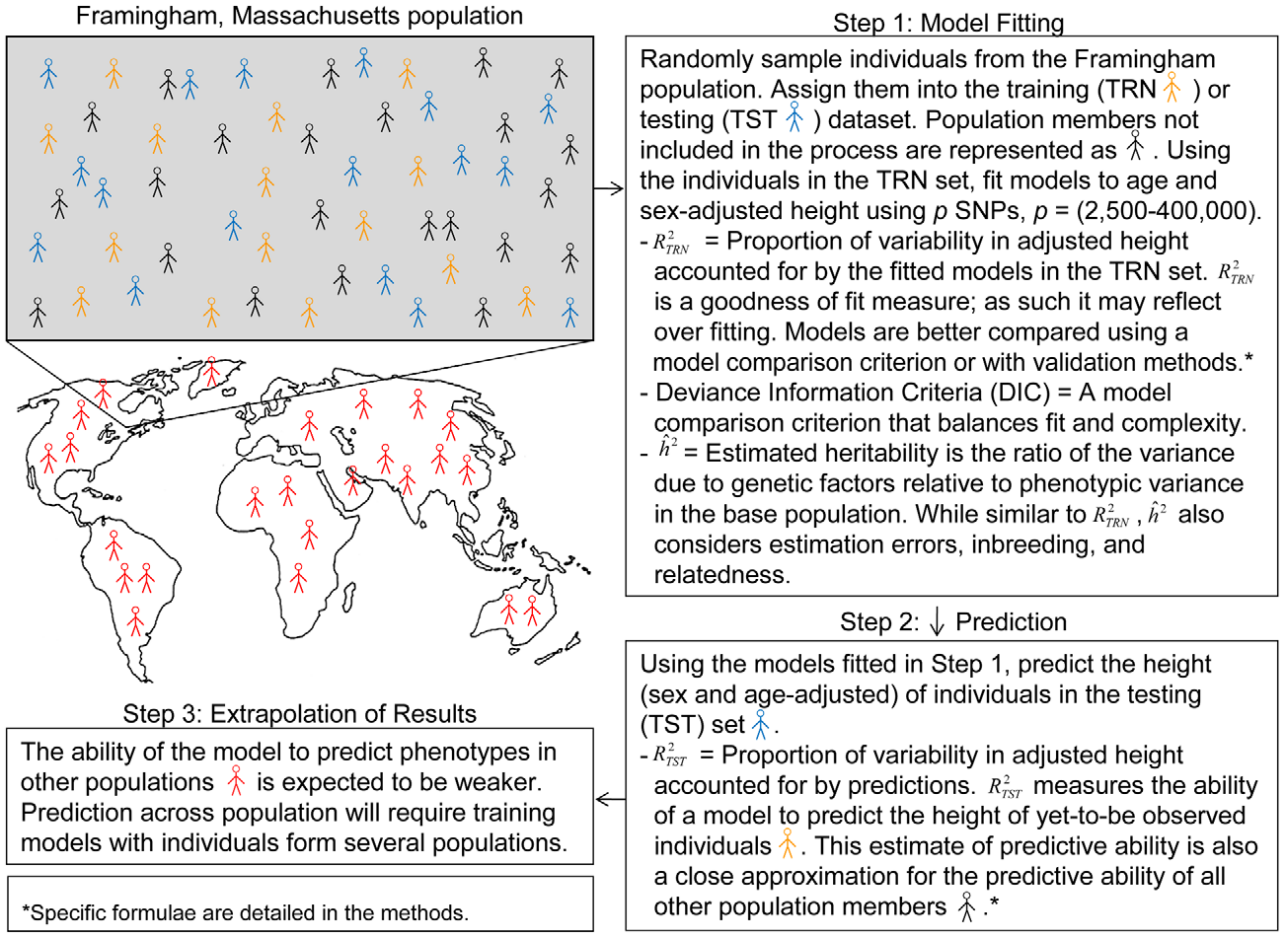
A simplified representation of assessment of goodness of fit in a training dataset and of predictive ability across a population: an example with the Framingham population.

The results of the Yang Study are particularly exciting due to their implications for eventual application to preventive and personalized medicine. However, a remaining question is the extent to which WGP methods improve the prediction of yet-to-be observed phenotypes, given the distinction between proportion of variance accounted for (as a measure of goodness of fit) and predictive accuracy (Figure 1). Heritability estimates can be regarded as measures of goodness of fit (see Materials and Methods for a discussion), yet it is well known that increasing goodness of fit will not necessarily lead to increased predictive accuracy in future samples, due to issues such as over-fitting [26]. In this study, we examine the relationship between estimates of variance accounted for and predictive ability using WGP methods. Using three different statistical approaches and validation designs, we examine how these relationships change as a function of the density of SNPs included, the size of the training sample, and the degree of familial information included in the training sample.

## Results

Using data from the Framingham Heart Study [27], [28], we built models for the age and sex adjusted height of 5,117 adults using between 2,500 and 400,000 SNPs. Participants included in our analyses were individuals greater than 18 years old from the original (N = 1,493) or the offspring (3,624) cohorts; 2,311 individuals were male and 2,806 were female. Height ranged from 141.6 cm to 198.1 cm with a mean of 167.4 cm (SD = 9.5 cm). Markers were incorporated into statistical models in two ways: (i) regression of adjusted height on marker genotypes via the Bayesian LASSO (BL) [29] ; (ii) Bayesian random effects models using a marker-based (realized) relationship matrix between individuals (G). There are multiple ways to map marker genotypes into G and none is considered generally superior. Here we considered those used by Hayes and Goddard [30] and the Yang Study; the two models are denoted as G^H^ and G^Y^, respectively, producing altogether three separate models. Goodness of fit was evaluated by means of the estimated residual variance and the proportion of variability accounted for by the fitted model in the training (TRN) dataset, 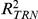. In addition, models were compared based on the estimated heritability, 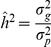 (where 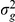 is the variance attributed to additive genetic effects and 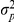 is the total phenotypic variance) and the Deviance Information Criterion (DIC) [31].

Table 1 gives the estimated 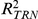, 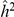, and DIC by model and number of SNPs. Both 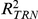 and 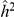 increase as more SNPs are included in the model, indicating an improved model fit. With 400,000 SNPs, the 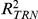 statistic indicates that predicted genetic values (see Materials and Methods for a detailed description of terminology) accounted for 95% of the variability in adjusted height (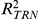), and the estimated heritability (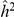
∼0.83) is close to what has been previously reported for this trait. Based on the trend observed, any further increases in common SNPs would likely produce a minimal increase in the proportion of accounted variability.

**Table 1:** R-squared statistic measured in the data used to train the model (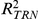), estimated posterior mean of heritability (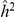), and Deviance Information Criterion (DIC) by model and number of SNPs (where K = 1,000).

Number of SNPs	Bayesian Lasso^1^		Genomic Relationship G^Y^			Genomic Relationship G^H^		
	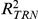	DIC	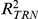	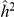	DIC	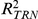	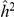	DIC
**2.5K**	0.33	32,920	0.36	0.21	32,883	0.34	0.26	32,912
**5.0K**	0.47	32,666	0.49	0.31	32,605	0.48	0.37	32,642
**10K**	0.65	32,106	0.69	0.47	31,950	0.66	0.52	32,081
**20K**	0.79	31,359	0.82	0.60	31,124	0.79	0.65	31,365
**40K**	0.87	30,564	0.89	0.70	30,201	0.87	0.74	30,564
**80K**	0.92	29,629	0.93	0.77	29,220	0.92	0.80	29,685
**160K**	-	-	0.95	0.79	28,925	0.93	0.81	29,416
**400K**	-	-	0.96	0.81	28,444	0.94	0.83	29,017

Estimates were obtained by fitting models to height adjusted by sex and age and using all available data (N = 5,117).

1 For the Bayesian LASSO, due to high memory requirements, only models including up to 80K markers were considered. This model does not include a genetic variance parameter, therefore it does not yield a direct estimate of heritability. For this reason heritability is not reported for this model.

As the number of markers increases, DIC decreases, indicating that information is continually being added to the model. This conforms with expectations under an infinitesimal model where the proportion of variance at Quantitative Trait Loci (QTLs) accounted for by regression on SNPs should increase with marker density [32]. Moreover, for any given number of SNPs, differences in the estimated residual variance, 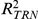, and heritability estimates across statistical approaches were small. We do not report 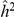 based upon the Bayesian LASSO: while formulae have been proposed to arrive at estimates of genetic variance from estimated marker effects and allele frequencies, they are problematic as they rely on the unrealistic assumption of linkage equilibrium between markers [33]. However, the similarity in 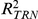 across models suggests that the proportion of variance accounted for by the Bayesian LASSO is similar to that of the two other methods.

To evaluate predictive ability, we used three different validation designs. *Approach A-* 10-fold cross-validation (CV) with assignment of individuals to folds at random. Because of the multiple generations present in the Framingham dataset, it is possible for children to be used to predict their parents in this design, which does not correspond to a standard prediction problem. To avoid this situation, we employed *Approach B*- using parents to predict children, we constructed a training dataset (TRN) with 1,493 parents and a testing dataset (TST) comprising offspring (N = 3,624). Because of the structure of the data, the size of the training sample used in Approach B is much smaller than that used in Approach A. Theory and empirical evidence [32] suggest that the accuracy of estimates of genetic values depends on the size of the training sample. To explore how much the size of the training sample affects predictive ability, we devised *Approach C*- randomly split the sample 10 times into TRN (N = 1,493) and TST sets (N = 3,624). Therefore, Approaches B and C differ in the way individuals were assigned to TRN and TST sets but not on the size of the TRN set. While approaches A and C allow for replicate datasets (10 in this study), Approach B is constrained to one replicate. As an aside, replicate datasets yielded highly similar 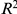 values, with an average coefficient of variation of <0.5%.

**Figure 2:**
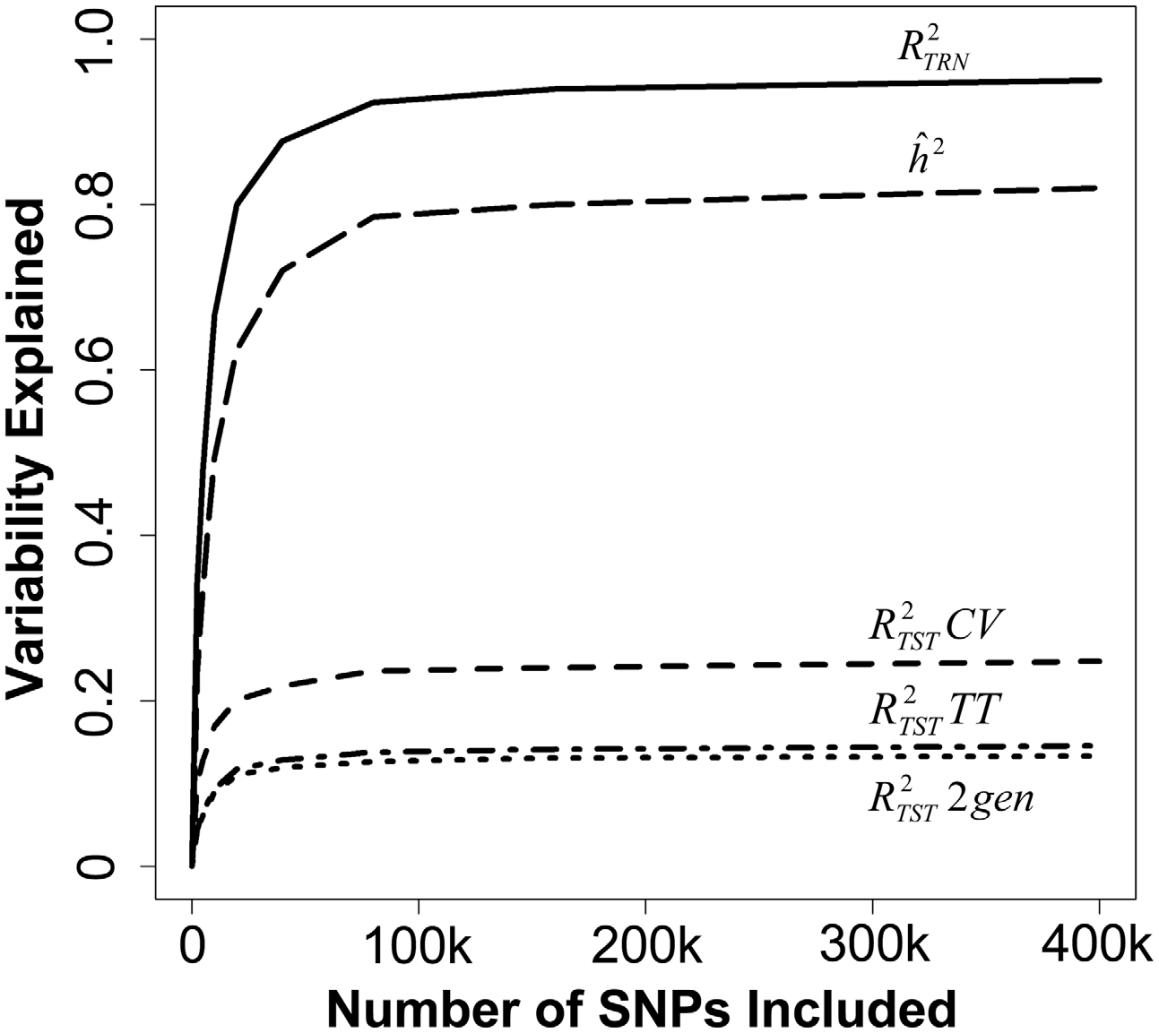
We averaged the estimates of 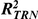 (measured in the training data), 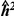, 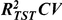 (measured in a 10 fold cross validation), 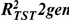 (measured in a 2 generation validation), and 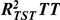 (measured in a replicated Training-Testing validation) over the three modeling techniques (BL, G^H^, G^Y^) and showed their relationship to the number of SNPs included in the model.

Table 2 displays the estimated 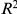 evaluated in validation (TST) samples (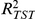) by model, validation design, and number of SNPs. Within all validation designs, differences between models were very small. To better visualize the relationship between 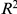, 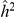, and the number of SNPs, we average the results across modeling techniques (Figure 2). Predictive accuracy increased with the number of SNPs, reaching an 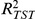 of 25% in the 10-fold CV design when 400,000 SNPs were used. In the other two validation designs (approaches B and C), 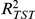 is considerably smaller than in the 10-fold CV, reaching a maximum 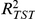 of 13% (15%) in the 2-generation and random training-testing designs, respectively. The 10-fold CV uses larger relative training datasets than approaches B and C, which can affect prediction accuracy in at least two ways. First, using larger training datasets is expected to increase accuracy, even with nominally un-related individuals [32]. Concurrently, when the size of the training dataset is increased, the likelihood of having multiple close relatives included in the training data also increases, and, as we discuss below, for a fixed sample size, prediction accuracy increases with the number of close relatives used to train the model. Unfortunately, the CV designs we evaluate do not allow exact separation of the relative effect of sample size from that of other contributing factors.

**Table 2:** R-squared between predicted and observed values (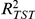) estimated using different number of SNPs (where K = 1,000), models, and validation designs.

Number of SNPs	10-Fold CV^1^			2-Generations design^2^			Training-Testing Random^3^		
	BL	G^Y^	G^H^	BL	G^Y^	G^H^	BL	G^Y^	G^H^
**2.5K**	.097	.102	.098	.054	.035	.035	.064	.035	.033
**5.0K**	.126	.130	.129	.066	.058	.061	.080	.059	.057
**10K**	.166	.174	.168	.087	.088	.093	.099	.094	.088
**20K**	.200	.204	.199	.106	.111	.115	.119	.119	.114
**40K**	.217	.221	.216	.117	.118	.123	.128	.131	.126
**80K**	.236	.237	.236	.124	.126	.129	.138	.139	.137
**160K**	-	.240	.240	-	.130	.132	-	.142	.141
**400K**	-	.247	.249	-	.133	.133	-	.146	.145

BL = Bayesian LASSO, G^H^ = Goddard-Hayes, and G^Y^ = Yang study (see Materials and Methods for elucidation).

1 10-fold cross validation, where the training set comprised 4,605–4,606 individuals.

2 Models were trained using the original cohort (N = 1,493) and predictive ability was assessed in the Offspring cohort (N = 3,624).

3 Data was assigned at random to a training set (N = 1,493) and predictive ability was evaluated in the remaining individuals (N = 3,624). This was repeated 10 times; each time individuals were randomly assigned into training/testing sets. Results are averaged across the ten replicates.

The predictive accuracy of WGP methods is known to depend on how closely related individuals in the training and validation samples are to each other [34]–[36]. The Framingham Heart Study dataset contains varying degrees of familial relationships (e.g., parents, offspring, and siblings) and provides the opportunity to study how prediction accuracy is affected by including familial members in the training population. To demonstrate this effect, for every individual in the 10-fold CV testing datasets, we calculated the number of close relatives (parents, full sibs, half sibs and offspring) present in the training dataset used to derive its prediction. This was calculated as follows: let 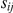 be an index which takes the values of 1 if individuals 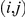 are either full sibs or a parent-offspring pair, 0.5 if 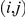 is a half-sib pair, or 0 otherwise. Using this system, a score was calculated as 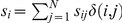 where 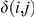 equals one if individual *i* is in the testing population and individuals *j* is in the training population, and zero otherwise. Using this score we classified individuals into four groups (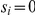, 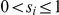, 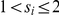, 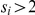) and calculated the average 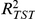 within each group after pooling the groups across CV folds.

**Figure 3:**
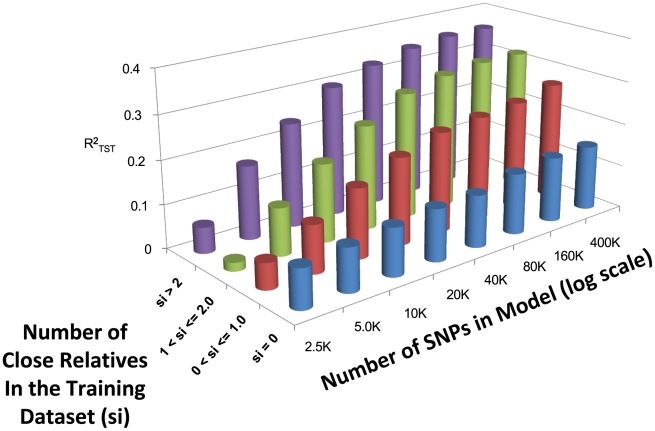
Averaged (across the three different models) estimates of 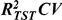 (measured in a 10 fold cross validation) while varying the number of close relatives (s_i_) in the training dataset with 2.5K to 400K SNPs.

Figure 3 depicts the relationship between the number of close relatives in the training population, the number of SNPs, and 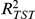 averaged across the three modeling techniques (see Table S1 for exact performance values). As expected, when the number of close relatives in the training dataset increases, the predictive ability increases. The relative increase in predictive ability with increasing SNP density is dependent upon the number of close relatives included in the model, with more drastic increases in predictive ability observed when more than two close relatives are included within the training dataset. When 400,000 SNPs are included, the average 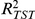 is 0.154, 0.267, 0.322, and 0.363 when 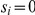, 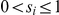, 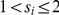, and 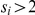, respectively.

## Discussion

Our results are concordant with the Yang Study, demonstrating that much of the variance in human height can be accounted for using WGP methods based on common SNPs. However, there are a number of differences between our studies that warrant consideration. First, we focused on prediction accuracy and several factors that may affect it, while the Yang Study focused on estimating the proportion of variance in human height that can be explained by common SNPs. While we report heritability estimates, we stress that our estimates of 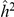 are not comparable to the 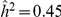 reported by the Yang study because, unlike the Yang Study, we did not restrict our sample to be composed of nominally unrelated individuals. While removing related individuals may allow estimation of genetic variance solely attributable to common SNPs through LD with causative polymorphisms, the use of exclusively un-related individuals may harm a model's ability to separate genetic signal from non-genetic components [36] and therefore measures of prediction accuracy derived from such approach may under-estimate the predictive power of common SNPs. In addition, we focused on adult height (≥18 years old), while the Yang Study included individuals ≥16 years of age, which may induce added non-genetic variability as some teenagers will still be growing at that age. Finally, there likely are differences between the Framingham population and the Australian population used in the Yang study.

In all validation designs, we found that predictive ability increased with the number of SNPs, suggesting that a large number of SNPs are needed to capture genetic variance at QTLs. These results are similar to findings in the animal breeding literature for infinitesimal traits [37], [38]. Our results also suggest a diminishing rate of return, with the difference in predictive ability between 80,000 and 400,000 SNPs being only ∼6% in the 10-fold CV. However, the number of markers at which this “plateau” occurs is likely to depend on multiple conditions such as the extent of LD in the population and the number of individuals in the training data. Indeed, other studies using populations with smaller effective population sizes (N_e_), and therefore larger LD spans, have reported high accuracy with much sparser coverage [37], [38].

A recent study [39] reported a decrease in predictive ability of human height for models with p-value inclusion thresholds greater than 5×10^−3^; suggesting that prediction accuracy may be harmed by including a large number of markers in a predictive model. However, an important difference between this study and ours is that in the former, marker effects were estimated using a fixed effects model while we use a Bayesian mixed model framework where all unknowns are modeled as random effects. Unlike the fixed effects approach, the Bayesian mixed model framework induces a shrinkage of estimates which, to some extent, controls over-fitting and seems to prevent a reduction in predictive ability in models with p≫n.

Importantly, we found no drastic differences between any of the statistical methods we considered. This is not surprising given that all three methods are based on an underlying additive model and that height likely conforms to an infinitesimal architecture. Moreover, these results are in agreement with findings reported in the animal breeding literature [40] which report small differences in predictive ability between contrasting methods. However, this conclusion may not apply to traits with simpler architecture, e.g., traits where major associated variants explain a substantial proportion of genetic variance. In these cases, models using marker-specific shrinkage of estimates such as the BL may outperform models such as G^H^ or G^Y^ where all markers are equally weighted.

Theoretical [32], [41] and empirical studies [37], [38] demonstrate that prediction accuracy increases monotonically with the size of the training population. Our results showed the same pattern, with a ∼70% increase in predictive ability when the size of the training dataset was increased from 1,493 to 4,506. A practical question resulting from this is how many individuals are needed to attain a certain predictive accuracy. The answer to such question depends on several factors such as trait heritability, marker density, N_e_, the genetic architecture of the trait, and the degree of propinquity between individuals whose phenotypic outcomes are to be predicted and those used to train the model. For nominally unrelated individuals under an infinitesimal model for a trait with *h^2^* = 0.8, Goddard and Hayes [41] report that for effective population sizes of 100 or 1,000, achieving a correlation between predicted and true genetic values of 0.7, or equivalently, an 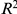 between predicted and realized height of about 0.39 (calculated as 0. 7^2^×0.8 ), requires training samples of approximately 4,000 and 50,000 individuals, respectively. However, as our results illustrate, prediction accuracy can be increased substantially by using information from related individuals.

Simulation [34] and empirical studies [35], [36] in animal breeding have suggested that the prediction accuracy of WGP methods depends on familial relationships between individuals in the training and validation samples. This was confirmed by our analysis: in the 10-fold CV with 400K SNPs, the 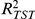 of individuals whose prediction was derived without using information from close relatives in the training dataset (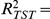 0.15) is much smaller than that obtained when direct relatives were included in the training dataset (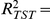 0.27, 0.32, and 0.36, for individuals with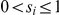, 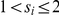, and 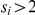 respectively). This occurs because WGP methods exploit genetic similarity across individuals and because recent family history plays a central role in determining genetic similarity. In light of this observation, one may wonder: does the use of genetic markers simply recapitulate pedigree-relationships? Several studies in animal and plant breeding have demonstrated the superiority of WGP over pedigree methods [40], [42]–[44] suggesting that markers convey more information than that provided by pedigrees. In particular, molecular markers can account for similarity/differences due to common ancestry not traced by the pedigree, and, more importantly, markers can account for differences due to Mendelian segregation. Relative to plant or animal breeding populations, the level of inbreeding in humans is smaller, with the quality of pedigree information typically being poorer, if it is even available. Therefore, the benefits of using markers relative to pedigree information for prediction could be even larger in humans.

Clearly, there exists a redundancy between the information conveyed by the pedigree and that provided by markers. However, this redundancy is not complete and there may be benefits to incorporating pedigree and marker information in the model. For example, Vazquez et al. (2010) used data from US Holsteins to quantify the prediction accuracy using pedigree-based predictions, marker based WGP, and predictions combining pedigree and markers. The study confirmed the superiority of marker-based models (with a correlation of 0.42 for pedigree-based predictions and 0.649 for the marker-based predictions in CV) and found that, when more than 10,000 markers were available (for a Holstein sample), combining pedigree and molecular marker data was no better than using marker data only. This suggests that dense markers are able to capture genetic similarity due to recent family history as well as other sources of genetic similarity not described by pedigrees. Therefore, we speculate that the largely incomplete pedigrees of most humans will provide little to no additional information for the prediction of complex traits, especially given the high density of markers typically available.

A pertinent question is whether a WGP model fitted to one population can be used to predict phenotypes in a distantly related population; this remains, so far, an un-answered question [14]. The prediction accuracy of WGP methods depends on the patterns of LD between markers and QTLs; these are likely to change across populations and therefore it is reasonable to expect relatively poor prediction accuracy across populations. This does not represent a failure of the methodology per se, but instead a feature that needs to be considered when applying these methods for prediction.

Population structure, admixture, or other population features can lead to spurious associations and affect prediction accuracy; therefore accounting for these features has been an important focus for GWAS analyses [45]. A pertinent question is the extent to which structure and other forms of genetic diversity are accounted for by WGP methods. An important difference between WGP methods and standard single-marker regressions is that, when all markers are jointly modeled, population structure, admixture, familial relationships, genetic differences between full-sibs within a family, and genetic relationships between nominally un-related individuals are all implicitly accounted for to the extent that whole-genome markers describe them. Indeed, regressing a phenotype simultaneously on a set of whole-genome markers is equivalent to regressing the phenotype on all marker-derived principal components, with a degree of shrinkage in the estimated effect for each component that is proportional to its associated squared-singular value [46]. The Framingham population consists of individuals from various European ethnic backgrounds and height is typically correlated with northern European ancestry; therefore, population stratification is likely contributing to prediction accuracy [47]. Conversely, the patterns of LD between markers and QTL may be different across sub-populations and this may hinder predictive ability, especially when the sub-populations were separated for many generations [48]. The exact nature of this tradeoff is difficult to establish and constitutes an important area of future exploration.

In conclusion, WGP methods provide a promising approach for the prediction of complex traits. The results of the Yang Study and those reported in this study both support this conclusion: they account for a larger proportion of the expected genetic variance and, as our study indicates, are able to predict yet-to-be observed phenotypes with greater success. Yet, it is apparent that predictive ability depends to a large part upon how many close relatives are included while training the model, and there is an apparent need for improving the accuracy of predictions of nominally unrelated individuals. Therefore, while whole-genome prediction of complex human traits can yield more accurate predictions than those based on models using a reduced number of markers, prediction of such traits remains difficult and significant room for improvement exists.

## Materials and Methods

### Genotyping and Quality Control

Subjects were genotyped using the Affymetrix GeneChip Human Mapping 500K Array Set. For details on genotyping, see http://www.ncbi.nlm.nih.gov/projects/gap/cgi-bin/study.cgi?study_id=phs000007.v3.p2. SNPs with call rates less than 90% and with a minor allele frequency (MAF) less than 3% were excluded. The remaining missing genotypes were imputed by sampling from a Binomial distribution using the empirical MAF estimate under the assumption of Hardy-Weinberg Equilibrium.

### Genome-Wide Models for Human Height

In all models, age and sex-adjusted height of individual *i*, 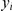, was expressed as 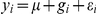 where: 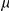 is an effect common to all individuals, 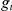 is a genetic value (i.e., a component of phenotypes that can be attributed to genetic factors), and 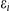 is a model residual which captures all factors affecting the response not captured by 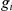. The conditional distribution of the data is:
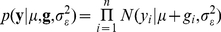
(1)where, 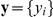, 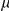 is an effect common to all individuals, **g** = {*g_i_*} is a vector of genetic values, and 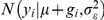 is a normal density for the random variable, 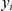, centered at 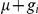, with variance 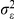.

All models were implemented in a Bayesian framework with inferences based on the posterior distribution of the unknowns given the data. Models differed in the number of markers used and the way they were incorporated into 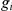. In the first group of models, genetic values were assumed to be multivariate normal:
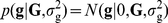
(2)where 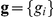, 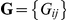 is a relationship matrix between individuals *i,j* computed from marker genotypes and 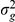 is an additive variance parameter. This approach has been used in many applications for modeling infinitesimal additive effects using molecular markers [12], [30], [49]–[51]. We focus on those used by Hayes and Goddard [30] (G^H^) and the Yang Study (G^Y^) to generate **G** from the marker data. In method G^Y^, relationships are standardized so that the average diagonal value equals one. In order to make the genetic variance parameters comparable, this standardization was also applied to G^H^ by dividing the entries of **G** by the average diagonal value.

To estimate the remaining model parameters, we utilized a Bayesian approach by assigning prior distributions to 
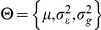
. We assigned a flat uniform prior to 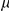, with conjugate scaled inverse chi-square priors used for 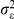 and 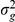, implying a joint posterior density proportional to:
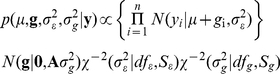
(3)Samples from the posterior distribution of the above model were obtained using a Gibbs sampler implemented in the R-language (http://www.R-project.org). We specified the hyper-parameters in [3] as 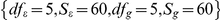. These values give a prior expectation of the variance of genetic values and of model residuals that are equal to approximately one half of the sample variance of adjusted height. With 5 degrees of freedom, priors have finite mean and variance, and a relatively small influence on inference.

In a third model, genetic values were described as a linear regression on marker covariates: 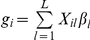. Here, 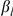 is the additive effect of the *l^th^* marker. Marker effects were inferred using the Bayesian LASSO (BL) of Park and Casella [29]. This model has been used successfully to model complex traits in genetic applications [37], [43], [52]. This leads to the joint posterior distribution density:
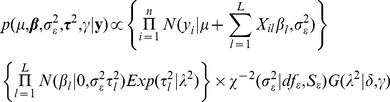
(4)where 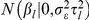 denotes a normal prior assigned to 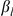 centered at zero and with prior variance equal to 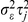, 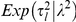 is an exponential prior assigned to the 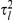
's , and 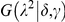 is a Gamma prior assigned to the regularization parameter 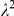. This model was fitted using the BLR package [53] in R. The use of SNP-specific conditional prior variances, 
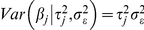
, allows for SNP-specific shrinkage of the estimates of effects. This contrasts with models G^H^ and G^Y^ in which all markers are equally weighted. The joint posterior distribution given by [4] is indexed by several hyper-parameters. In our application, those hyper-parameters were: 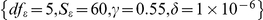. These values give a prior expectation of the residual variance that is about one half of the sample variance of adjusted age and a relatively flat prior density over a wide range of the regularization parameter 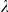. We applied the above-mentioned models using subsets of evenly-spaced SNPs, ranging from 2,500 to 400,000. Due to limitations in RAM-memory, the maximum number of SNPs considered for the BL (method 3) was 80,000.

### Heritability and R-Squared

Heritability, 
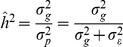
, is defined as the ratio of the variance due to additive genetic factors, 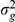, relative to the phenotypic variance, 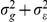, in the base population (in a pedigree-model, this is the population from where the founders were sampled, which is assumed to be comprised of un-related individuals). This is also the squared correlation between genetic values and phenotypes, and the proportion of variance accounted for by genetic factors, both in the base population [54]. Heritability *estimates* (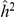) are commonly obtained by replacing population parameters with estimates derived using Restricted Maximum Likelihood or Bayesian procedures.

The 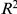 statistic is the ratio between the variance accounted for by a model relative to the sample variance of the response. That is: 
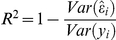
 where 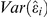 is the sample variance of predictive residuals derived from a model and 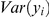 is the sample variance of phenotypes. The 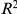 statistic is related to 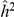. However, 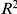 measures the proportion of variance accounted for by predicted genetic values in the sample, while 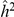 estimates the proportion of phenotypic variance accounted by true genetic values in the base population. Fundamentally, 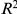 ignores inbreeding, relationships between individuals in the sample and estimation errors; therefore, it is not a consistent estimate of heritability [54], [55].

The 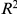 statistic is sometimes evaluated in the same dataset that was used to derive predictions, which tend to over-estimate predictive ability. A better assessment of the ability of a model to predict future data can be obtained using validation methods [26]. We therefore distinguish two R-squared measures: 
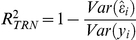
 and 
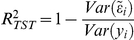
 where: 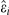 denotes a prediction error derived when all available data, including the *i^th^* observation, was used to fit the model, and 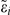 denotes a prediction error derived when the validation set containing the *i^th^* observation was not used to fit the model, respectively. Therefore, 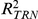 measures goodness of fit between the training data and the model while 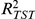 measures the ability of the model to predict future observations.

## Supporting Information

Table S1R-squared between predicted and observed values (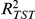) estimated using different number of SNPs with different numbers of relatives in the training populations averaged across validation designs.(DOC)
