# Supplementary material for: Beyond Missing Heritability: Prediction of Complex Traits
Source: PLoS Genet. 2011 Apr 28;7(4):e1002051. doi: 10.1371/journal.pgen.1002051 (PMC3084207; doi:10.1371/journal.pgen.1002051)
Supplement: Table S1 — R-squared between predicted and observed values () estimated using different number of SNPs with different numbers of relatives in the training populations averaged across validation designs. (DOC) [file pgen.1002051.s001.doc]

Table S1. R-squared between predicted and observed values ($R_{TST}^{2}$) estimated using different number of SNPs with different numbers of relatives in the training populations averaged across validation designs.

| **Number of SNPs** | **Number of relatives in training sample** | | | |
| --- | --- | --- | --- | --- |
|  | **0** | **0.5 – 1.0** | **1.5 – 2.0** | **> 2** |
| **2.5K** | 0.09 | 0.06 | 0.02 | 0.06 |
| **5.0K** | 0.10 | 0.11 | 0.11 | 0.17 |
| **10K** | 0.11 | 0.16 | 0.18 | 0.24 |
| **20K** | 0.12 | 0.20 | 0.24 | 0.30 |
| **40K** | 0.12 | 0.23 | 0.29 | 0.33 |
| **80K** | 0.14 | 0.24 | 0.31 | 0.35 |
| **160K** | 0.15 | 0.25 | 0.32 | 0.36 |
| **400K** | 0.15 | 0.27 | 0.32 | 0.36 |
